# Treatment outcomes of drug susceptible Tuberculosis in private health facilities in Lagos, South-West Nigeria

**DOI:** 10.1371/journal.pone.0244581

**Published:** 2021-01-20

**Authors:** Olanrewaju Oladimeji, Victor Adepoju, Felix Emeka Anyiam, James Emmanuel San, Babatunde A. Odugbemi, Francis Leonard Mpotte Hyera, Maureen Nokuthula Sibiya, Sanni Yaya, Ayuba Ibrahim Zoakah, Lovett Lawson

**Affiliations:** 1 Department of Public Health, Walter Sisulu University, Eastern Cape, South Africa; 2 Faculty of Health Sciences, Durban University of Technology, Eastern Cape, South Africa; 3 Faculty of Medicine, Department of Family Medicine and Public Health, University of Botswana, Gaborone, Botswana; 4 Adolescent Friendly Research Initiative and Care (ADOLFRIC), Ekiti State, Nigeria; 5 Center for Health and Development, University of Port Harcourt, Port Harcourt, Nigeria; 6 Kwazulu-Natal Research and Innovation Sequencing Platform (KRISP), College of Health Sciences, University of KwaZulu-Natal, Durban, South Africa; 7 Department of Community Health and Primary Health Care, Lagos State University, College of Medicine, Ikeja, Lagos, Nigeria; 8 School of International Development and Global Studies, University of Ottawa, Ottawa, Canada; 9 The George Institute for Global Health, The University of Oxford, Oxford, United Kingdom; 10 Department of Community Medicine, Jos University Teaching Hospital, University of Jos, Plateau State, Nigeria; 11 Zankli Medical Services Ltd, Utako District, Abuja, Nigeria; 1. IRCCS Neuromed 2. Doctors with Africa CUAMM, ITALY

## Abstract

**Background:**

The Lagos State Tuberculosis, Buruli Ulcer, and Leprosy Control Program (LSTBLCP) started engaging private hospitals under the Public-Private Mix (PPM) Program in 2008. The study aimed to evaluate the trend and predictors of successful Tuberculosis (TB) treatment outcomes of patients managed across these private health facilities between 2010–2016 in Lagos, Nigeria.

**Methods:**

Retrospective review of TB treatment register and treatment cards of patients commenced on TB treatment between January 2010 and December 2016 in 36 private health facilities engaged by the LSTBLCP. Between December 2016 and February 2017, data were collected and entered into Microsoft Excel by trained data entry clerks. The analysis was done using SPSS software. Independent predictors of successful treatment outcomes were determined using multivariate analysis at the statistical significance of p<0.05 and 95% confidence interval.

**Results:**

A total of 1660 records of TB patients were reviewed. 1535 (92.47%) commenced treatment, while 1337 (87.10%) of all records had documented treatment outcomes. Of the 1337 patients with outcomes, 1044 (78.09%) had a successful treatment outcome, and 293 (21.91%) had an unsuccessful outcome. Majority were male, 980 (59.04%), Human Immunodeficiency Virus (HIV) negative status, 1295 (80.24%), diagnosed with smear, 1141 (73.14%), treated in private not-for-profit (PNFP) hospital, 1097 (66.08%), treated for TB between 2014–2016 (18.96%-19.52%). In multivariate analysis, age>20years (aOR = 0.26, p = 0.001), receiving TB treatment in 2013 (aOR = 0.39, p = 0.001), having genexpert for TB diagnosis (aOR = 0.26, p = 0.031) and being HIV positive (aOR = 0.37, p = 0.001) significantly reduced likelihood of successful treatment outcome. The site of TB, being on ART or CPT, were confounding determinants of successful treatment outcomes as they became non-significant at the multivariate analysis level.

**Conclusion:**

Treatment outcome among Lagos private hospitals was low compared with NTBLCP and World Health Organization (WHO) target. We urge the government and TB stakeholders to strengthen the PPM interventions to improve adherence, particularly among People Living with HIV (PLHIV) and older TB patients. Hence, promotion of early care-seeking, improving diagnostic and case holding efficiencies of health facilities, and TB/HIV collaborative interventions can reduce the risk of an unsuccessful outcome.

## Introduction

Nigeria is one of the 30 high burden countries for tuberculosis (TB), TB/HIV, and Multidrug-Resistant TB (MDR-TB) globally [1]. Globally, an estimate of ten million people was reported to have been infected with TB in 2017. In 2018, TB incidence was 219/100,000 population, and it was estimated that 407,000 developed TB with barely 100,000 notified amouting to treatment coverage of 24% [2]. Tuberculosis (TB) is also the leading cause of death from infectious diseases in Nigeria [3]. Globally, the World Health Organization reports that about 5,000 TB related deaths are recorded daily, of which, 95% occur in low- and middle-income countries [3]. The World Health Organization (WHO) introduced a standardized, directly observed treatment, short-course (DOTS), and Stop TB Strategy to scale up TB prevention and control. These initiatives were adopted by Nigeria in 1993 and 2006, respectively [4]. Despite the above initiatives, a significant number of people are unknown to the health system or are not receiving quality TB treatment. The majority of these belong to the private providers of health services. In 2017, the World Bank estimated that Treatment Success Rate (TSR) in Nigeria improved from 79% to 86% between 2000–2016 [5]. However, disaggregated TSR data in public and private sectors are often lacking in annual National Tuberculosis, Buruli Ulcer and Leprosy Control Program (NTBLCP) report while reported TSR figures at sub-national levels varied across public and private facilities in Nigeria [6,7]. Treatment gaps and poor quality of TB treatment persist at the facility levels [8].

Some of the challenges of successful TB treatment include the long duration of treatment which results in non-adherence to medications and unfavourable social determinants of health [9,10]. Social determinants of tuberculosis have important influence on treatment outcomes. For instance, previous studies found that low education, alcohol abuse and low income background are major socioeconomic determinants that contributed to increased risk of tuberculosis and its unsuccessful outcomes [10]. Non-adherence increases the risk of unsuccessful treatment outcomes, relapse, and drug-resistant strains [11]. Meeting the global target of successful TB treatment is critical to the common goal of ending TB. Since 2007, efforts have been made by the NTBLCP to expand the TB control program beyond the public sector to the private sector with the view to linking all healthcare providers to the NTBLCP surveillance systems [4]. However, this has been slow and not at the scale needed for national impact on the improved notification and successful outcomes. Thailand and the Philippines were early adopters of the Public-Private Mix (PPM). Previous studies from the Philippines and Thailand showed TSR figures of 79.5% and 67% respectively from the private sector [12,13]. Studies from India and Nepal also provided compelling evidence that engaging the private sector was cost-effective, increased notification, and successful treatment outcome [14,15]. An aggressive expansion to the private sector in Nigeria, therefore, became crucial as 60% of Nigerians patronize the private providers as the first point of care when sick [4].

In 2014, the World Health Organization launched the End TB Strategy to end TB by 2035 [16]. The second pillar of the End TB Strategy envisioned PPM and emphasized on the need for engagement of civil society, communities, and the public and private providers of health services in tuberculosis control. This recommendation was taken forward post-2015 with a rapid effort to engage the private healthcare providers (PHPs) in Nigeria. The NTBLCP strategic roadmap also integrated several activities aimed at strengthening the private sector performance in tuberculosis control [17]. In Lagos Nigeria, the number of private hospitals engaged by the Lagos State TB Control Program increased from 8 in 2008 to 103 in 2016 [18].

Contemporary studies in the early days of PPM in Nigeria reported treatment success rate of 83.7%, 86%, 85.7% and 83.5% from private hospitals in Kaduna, Anambra, Lagos and Ogun Nigeria, respectively [6,19–21]. These figures were all below the national TSR target of 90%. WHO recommended that treatment outcome analysis should be carried out every year at national and Local Government Area (LGA) levels [16]. A successful TB control program should match a high treatment success rate with lower incident DRTB cases and vice versa. Poor TB treatment outcome has serious consequences, including ongoing community transmission and development of drug-resistant mycobacterium tuberculosis. It is estimated that the prevalence of MDR TB is 4.3% among new TB cases and 25% among retreatment cases respectively [2]. Despite the relatively high reported overall national TSR over the years, DRTB case notification in Nigeria has increased from 21 in 2010 to 1,686 in 2016 and a further increase of 35% from 1,686 in 2016 to 2,286 in 2017 [2]. In addition, a WHO survey in Mexico showed that one-third of patients who died from TB were managed by private practitioners [20]. TSR data disaggregated by type of facility (public versus private) is lacking in Nigeria, making efforts to monitor the success of recent DOTS program expanded into private facilities difficult. There is also a lack of data to confirm previous concerns that many TB patients managed in the private facilities were poorly managed and that private facilities contributed to increasing DRTB cases. Analysis of trends and predictors of TSR specifically for the private sector would help to test any ‘dilution effect’ in reported national TSR figures and focus interventions on unique factors influencing the unsuccessful outcome of patients managed for TB in the private sector. It is therefore pertinent to evaluate if treatment outcome in the private sector has improved or otherwise in recent years. The study assessed the trend in TB treatment outcome and its predictors among patients treated in Lagos private facilities between 2010 and 2016.

## Methods

### Study setting

Lagos has a population of over 22 million and houses 11% of the Nigerian population. The state is divided into 20 Local Government Areas (LGAs) [18]. TB surveillance data from engaged health facilities in the LGAs are collated by the LGA TB supervisors and reported to the State TB and Leprosy Control Officers, who in turn report to the NTBLCP. The engagement of private health facilities for TB control by LSTBLCP commenced in 2008 and increased from an initial eight to 103 engaged private health facilities by 2016. The LSTBLCP role includes an initial assessment of PHPs for infrastructure, client load, staffing, and willingness; training of staff and supplies of diagnostic equipment and drugs for eligible PHPs based on assigned service schemes, all at no cost [8]. Engaged PHPs are expected to provide TB patients with free anti-TB medications, although reasonable fees could be charged for consultation, registration, and microscopy services. Each of the 36 states in Nigeria has the State TB and Leprosy Control Program overseen by the NTBLCP to whom state TB data is submitted on quarterly basis [8]. LSTBLCP usually engaged health facilities under four service schemes categorized as scheme 1 (presumptive referral only); 2 (diagnosis only), 3 (Directly Observed Short Course Therapy and scheme 4 (TB diagnosis and treatment) [8].

The engagement of private hospitals by NTP was voluntary and based on interest. In the initial stage, health facilities were assessed for TB infrastructure and staffing, among others. When eligible, a Memorandum of understanding (MoU) was signed between the eligible and interested PHPs and LSTBLCP. Interested PHPs nominated staff called DOTS officers who were trained as per the NTBLCP guideline. Staff turnover and attendant gaps in tuberculosis service were not uncommon in the private sector after the training. Refresher training was usually conducted every 2–3 years, depending on the availability of funds. After the DOTS workshop; the facilities were subsequently engaged in any of the above TB service schemes. NTBLCP provided equipments like microscopy, anti-TB medications, laboratory reagents and consumables, recording and reporting tools, and other materials needed for a smooth takeoff. PHPs were expected to provide TB services at no cost. However, they were allowed to charge patients for consultation and registration and agreed discounted fee for smear diagnosis as embedded in the MOU.

### Study design

This was a retrospective review of TB register and records of individuals managed for TB across 36 private hospitals between January 2010 and December 2016. A purposive selection of 36 (high volume) out of a total of 103 private facilities from 13 high burden Lagos LGAs. We utilized records of all TB patients enrolled in TB treatment across select facilities. A total of 1660 participants were ultimately recruited for the study. The sample size was reasonably powered to detect differences between groups with successful and those with unsuccessful treatment outcomes.

### Inclusion criteria

Included in the study were patients of all age groups who were commenced on TB treatment in any of the 36 private health facilities between 2010–2016; having spent not less than six months on treatment during the review period; the facility was private and engaged by the LSTBLCP to notify TB cases; have been engaged not less than one year and have notified at least one TB case since the engagement. Patients diagnosed with MDRTB in these facilities; who did not have a definitive treatment outcome or who did not meet any of the above inclusion criteria were excluded from the study.

### Diagnosis of TB patients in the private health facilities

Prior to 2015, genexpert was restricted to the diagnosis of suspected TB/HIV co-infection, suspected multidrug-resistant TB cases, and relapse. By 2015, genexpert was adopted by the country as the first and preferred method of diagnosis for all forms of TB while smear diagnosis was limited to geographic areas where genexpert was not available. Genexpert was however used for follow up investigation [8]. In 2016, genexpert coverage was poor and not available in all the 20 Lagos LGAs. Patients sputum samples were sent from private health facilities to peripheral public facilities where genexpert were avaialble through the support of sputum transporters.

### Treatment of TB patients in private facilities

Tuberculosis medications were given to patients free-of-charge in private hospitals for the duration of treatment. Just like the public sector, patients treated by PHPs engaged by LSTBLCP managed all drug-susceptible TB patients for six months using a fixed-dose regimen (Rifampicin, Isoniazid, Pyrazinamide, and Ethambutol) except osteoarticular TB and TB meningitis which was treated for 12 months. Cases of drug-resistant TB were not managed by PHPs but referred to designated MDR treatment centre.

### Outcomes definition

#### Cured

A pulmonary TB patient with bacteriologically confirmed (smear or culture-positive)Tuberculosis at the beginning of treatment and who was smear or culture-negative in the last month of treatment and on at least one previous occasion.

#### Treatment completed

A TB patient who completed treatment but without any evidence of cure or failure (there is no record to show that sputum smear or culture results in the last month of treatment and on at least one previous occasion are negative either because they were not done or results were not available).

#### Treatment failure

A TB patient whose sputum smear or culture is positive at month five or later during treatment.

#### Died

A TB patient who dies for any reason before or during the course of treatment.

#### Lost to follow-up

A TB patient who did not start treatment or whose treatment was interrupted for two consecutive months or more.

#### Not evaluated

A TB patient for whom no treatment outcome is assigned. This includes cases “transferred out” to another treatment unit and where the treatment outcome is unknown to the reporting unit.

#### Treatment success

The sum of bacteriologically diagnosed TB cases cured and those who completed their treatment without a bacteriologically confirmed register.

#### Moved to 2^nd^-line treatment

Patients who became MTB detected/RIF Resistance detected at any point of their treatment and who are moved to CAT IV treatment.

### Measures

The primary outcome is a composite measure defined as successful treatment (Yes/No). Yes was assigned to TB treatment completed and cured, and No was assigned to all the other outcomes following the standard TB definition.

Independent variables comprised of the socio-demographic variables such as age, sex (male, female) and years; and the clinical variables such as facility type (private not for profit, private for-profit), site of TB (pulmonary, extrapulmonary), treatment regimen (category 1, category 2), a referral from the community (yes, no), DOTs by treatment supporters (yes, no), type of patients (new, others), method of diagnosis (microscopy, genexpert, cxray, others), HIV status (positive, negative, unknown), ART (yes, no), and IPT (yes, no).

### Data collection

Data were collected by six trained data clerks between December 2016 and February 2017. Data were first entered into Microsoft Excel transcopied from TB register and treatment cards. For each patient, data collected include age, sex, date of registration, type of patient, treatment category, sputum follow up results (months 2/3, 5 and 6), date of treatment initiation, and completion, antiretroviral therapy (ART) and cotrimoxazole therapy (CPT) status. We defined treatment category and patient type based on definitions in the 5^th^ edition of the NTBLCP workers manual.

### Data analysis

Data were entered into Microsoft Excel ^®^ version 2013, where it was coded and cleaned, and imported into the Statistical Package for Social Sciences (SPSS) statistical software version 20 for analysis. Descriptive statistics were employed to analyze categorical variables from respondent’s socio-demographic and clinical characteristics; and were presented as frequencies and percentages (%) in tables and charts. Inferential statistics were used to explore the association of the outcome and the independent variables using the bivariate logistic regression model. Multivariate logistics regression was used to control for confounding variables. All Odds Ratios (ORs) and adjusted Odds Ratios (aORs) were reported with their 95% CI, and a p-value ≤ 0.05 was considered statistically significant.

### Ethics

This study was approved by the National Health Research Ethics Committee of Nigeria (NHREC/01/01/2007). Permission was granted by the medical director of each private health facility. Participant information was anonymized and de-identified by assigning unique number before analysis to maintain confidentiality. As this was aroutinely collected program data, informed consent from the patients was not obtained. The named ethics committee approved the study and waived the need for consent.

## Results

### Socio-Demographic characteristics of participants

**Fig 1:**
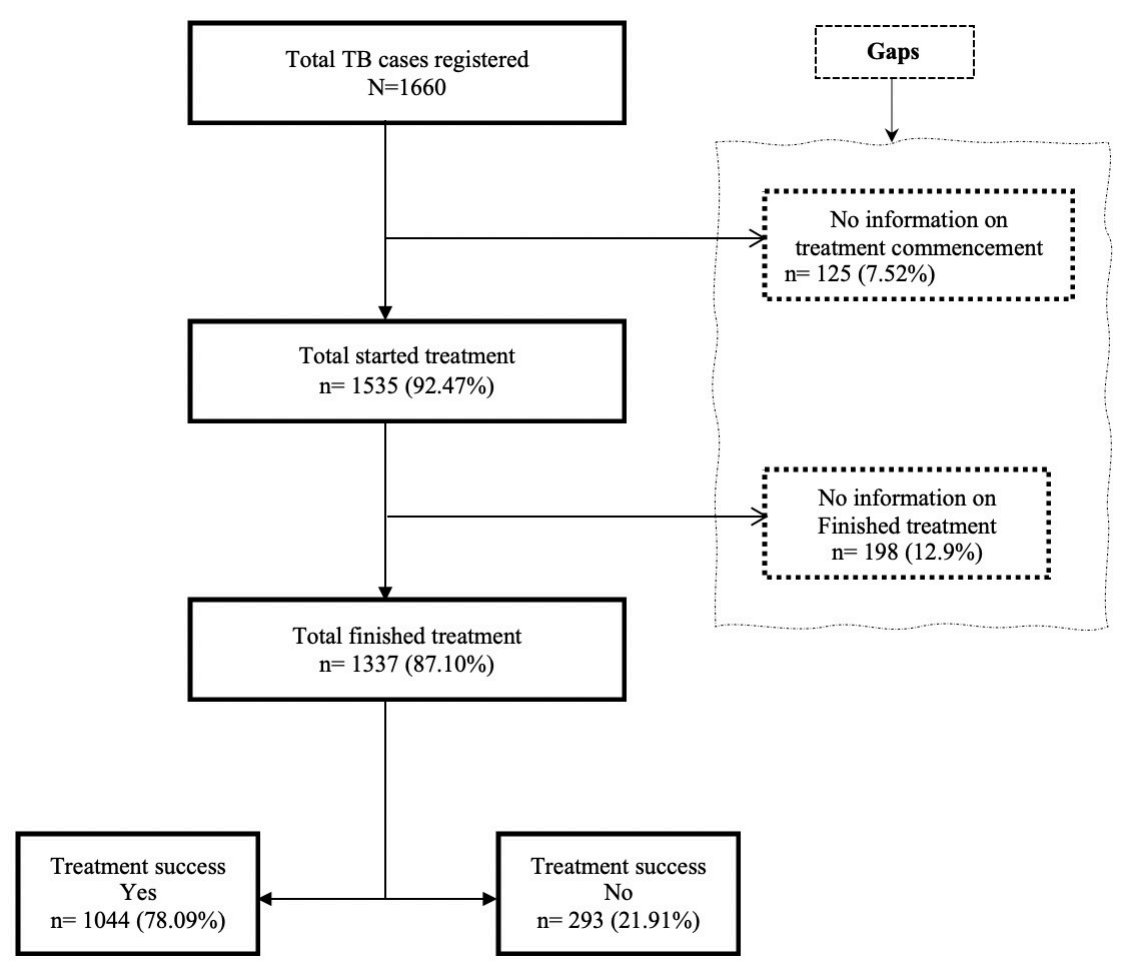
Flowchart of respondents registered for TB treatment in the private health facilities.

Overall, a total of 1660 participants were recruited for the study; 93.98% (1560) were tested for TB, 92.47% (1535) started treatment, and 87.10% (1337) completed treatment as shown in Fig 1.

The study participant’s within the age range 21–30 years had the highest proportion of 30.98% (513), followed by those within the age range 31–40 years, 26.99% (447). The least were those 60 years and over, 5.50% (91). Year of TB Diagnosis was observed to be the highest 2014–2016 (18.96%-19.52%) (Table 1).

**Table 1:** Demographics and clinical characteristics of respondent data (*n* = 1660).

Variables	Frequency	Percentage (%)
**Age (years) (n = 1656)**		
≤20	250	15.10
21–30	513	30.98
31–40	447	26.99
41–50	229	13.83
51–60	126	7.61
60+	91	5.50
**Year (n = 1609)**		
2010	51	3.17
2011	130	8.08
2012	218	13.55
2013	278	17.28
2014	305	18.96
2015	314	19.52
2016	313	19.45
**Sex (n = 1660)**		
Female	680	40.96
Male	980	59.04
**Facility Type (n = 1660)**		
Private not for profit (PNFP)	563	33.92
private for-profit (PFP)	1097	66.08
**Site of TB (n = 1622)**		
Pulmonary TB	1606	99.01
Extrapulmonary TB	16	0.99
**Treatment Regimen (n = 1632)**		
Category 2	54	3.31
Category 1	1578	96.69
**Patient referred from community (n = 686)**		
Yes	90	13.12
No	596	86.88
**DOTS by Treatment supporter (n = 1484)**		
Yes	1015	68.40
No	469	31.60
**Type of patient (n = 1594)**		
New	1511	94.79
others	83	5.21
**Means of diagnosis (n = 1560)**		
Acid-fast bacillus (**AFB**)	1141	73.14
Genexpert	100	6.41
Xray	168	10.77
Afb/genexpert	11	0.71
others	140	8.97
**Treatment Success (n = 1337)**		
Yes	1044	78.09
No	293	21.91
**HIV Status (n = 1614)**		
Negative	1295	80.24
Positive	130	8.05
Unknown	189	11.71
**On Anti-Retroviral Therapy (ART) (n = 87)**		
Yes	69	79.31
No	18	20.69
**On Co-trimoxazole preventive Therapy (CPT) (n = 35)**		
Yes	27	77.14
No	8	22.86

Most of the respondents were females, 40.96% (680), of which 33.92% (563) and 66.08% (1097) were from private for-profit (PFP) and Private not for profit (PNFP) facilities respectively. Most of the patients were managed for pulmonary TB, 99.01% (1606), on Category I regimen, 96.69% (1578) and did not get any form of a referral from the community, 86.88% (596) (Table 1).

Treatment supporter managed at the DOTS facilities was 68.40% (1015), with most of the patents being new, 94.79% (1511), and Acid-fast bacillus (AFB) being the most common means of diagnosis, 73.14% (1141), followed by chest X-ray, 10.77% (168). The treatment success rate was high among TB patients managed, 78.09% (1044), with most having an HIV negative status, 80.24% (1295), and a small proportion, 8.05% (130) having an HIV positive status. Among those that were HIV positive, most are on Anti-Retroviral Therapy (79.31%) and Co-trimoxazole preventive Therapy (77.14%) (Table 1).

### Factors associated with the number of TB treatments received among the respondents stratified by treatment outcome

From the bivariate logistics regression model (Table 2), significant associations for successful treatment outcome was found for the following variables: Age, year of TB diagnosis, Sex, Facility type, Site of TB, means of diagnosis, HIV status, on ART and on CRT.

**Table 2:** Factors associated with the number of TB treatments received among the respondents stratified by treatment outcome using bivariate logistic regression.

Variables	Successful Treatment Outcome		OR (95 CI)	*p-value*
	Yes (n = 1044)	No (n = 293)		
	Freq (%) (95CI)	Freq (%) (95CI)		
**Age (years) (n = 1656)**				
≤20 ^R^	177 (16.95)	31 (10.69)	*Ref*	
	[14.80–19.35]	[7.38–14.83]		
21–30	322 (30.84)	88 (30.34)	0.26(0.14–0.47)	*0*.001*
	[28.12–33.71]	[25.11–35.99]		
31–40	279 (26.72)	71 (24.48)	0.41 (0.24–0.68)	*0*.001*
	[24.13–29.49]	[19.64–29.85]		
41–50	145 (13.89)	36 (12.41)	0.38 (0.22–0.64)	*0*.001*
	[11.92–16.12]	[8.85–16.77]		
51–60	75 (7.18)	33 (11.38)	0.37 (0.21–0.66)	*0*.001*
	[5.77–8.91]	[7.96–15.61]		
60+	46 (4.41)	31 (10.69)	0.65 (0.35–0.66)	*0*.001*
	[3.32–5.83]	[7.38–14.83]		
**Year (n = 1655)**				
2010 ^R^	39 (3.74)	12 (4.11)	*Ref*	
	[2.74–5.07]	[2.14–7.07]		
2011	90 (8.62)	27 (9.25)	0.82 (0.39–1.70)	0.588
	[7.07–10.48]	[6.18–13.17]		
2012	183 (17.53)	27 (9.25)	0.80 (0.46–1.39)	0.419
	[15.34–19.95]	[6.18–13.17]		
2013	205 (19.64)	62 (21.23)	0.39 (0.23–0.67)	*0*.001*
	[17.34–22.16]	[16.68–26.38]		
2014	189 (18.10)	57 (19.52)	0.80 (0.51–1.26)	0.338
	[15.89–20.55]	[15.13–24.54]		
2015	224 (21.46)	64 (21.92)	0.80 (0.51–1.27)	0.340
	[19.07–24.05]	[17.31–27.11]		
2016	114 (10.92)	43 (14.73)	0.76 (0.48–1.19)	0.228
	[9.17–12.96]	[10.87–19.32]		
**Sex (n = 1660)**				
Female	453 (43.39)	103 (35.15)	*Ref*	
	[40.41–46.42]	[29.69–40.92]		
Male	591 (56.61)	190 (64.85)	1.41 (1.08–1.85)	*0*.012*
	[53.58–59.59]	[59.08–70.31]		
**Facility Type (n = 1660)**				
Private not for profit (PNFP)	420 (40.23)	71 (24.23)	*Ref*	
	[37.30–43.24]	[19.44–29.56]		
private for profit (PFP)	624 (59.77)	222 (75.77)	2.10 (1.57–2.83)	*0*.001*
	[56.76–62.70]	[70.44–80.56]		
**Site of TB (n = 1622)**				
Pulmonary TB	1013 (99.41)	277 (97.88)	*Ref*	
	[98.72–99.7]	[95.44–99.22]		
Extra pulmonary TB	6 (0.59)	6 (2.12)	3.66 (1.17–11.43)	*0*.026*
	[0.27–1.28]	[0.78–4.56]		
**Treatment Regimen (n = 1632)**				
Category 2	39 (3.81)	13 (4.50)	*Ref*	
	[2.80–5.17]	[2.42–7.57]		
Category 1	984 (96.19)	276 (95.50)	0.84 (0.44–1.59)	0.598
	[94.83–97.20]	[92.43–97.58]		
**Patient referred from community (n = 686)**				
Yes	52 (13.40)	15 (14.29)	*Ref*	
	[10.37–17.15]	[8.22–22.47]		
No	336 (86.60)	90 (85.71)	0.93 (0.50–1.73)	0.815
	[82.85–89.63]	[77.53–91.78]		
**DOTS by Treatment supporter (n = 1484)**				
Yes	663 (68.92)	168 (65.37)	*Ref*	
	[65.92–71.76]	[59.21–71.17]		
No	299 (31.08)	89 (34.63)	1.18 (0.88–1.57)	0.278
	[28.24–34.08]	[28.83–40.79]		
**Type of patient (n = 1594)**				
New	951 (94.91)	266 (94.66)	*Ref*	
	[93.37–96.11]	[91.35–96.98]		
others	51 (5.09)	15 (5.34)	1.05 (0.58–1.90)	0.868
	[3.89–6.63]	[3.02–8.65]		
**Means of diagnosis (n = 1560)**				
Acid-fast bacillus (AFB) ^R^	771 (78.27)	197 (72.43)	*Ref*	
	[75.59–80.74]	[66.71–77.65]		
Genexpert	13 (1.32)	7 (2.57)	0.62 (0.40–0.96)	*0*.031*
	[0.77–2.24]	[1.04–5.23]		
Xray	118 (11.98)	34 (12.50)	1.31 (0.48–3.56)	0.603
	[10.10–14.16]	[8.81–17.03]		
AFB/Genexpert	3 (0.30)	1 (0.37)	0.70 (0.40–1.22)	0.207
	[0.10–0.89]	[0.01–2.03]		
Others	80 (8.12)	33 (12.13)	0.86 (0.81–8.05)	0.856
	[6.57–9.99]	[8.50–16.61]		
**HIV Status (n = 1614)**				
Negative ^R^	857 (82.09)	192 (65.53)	*Ref*	
	[79.65–84.30]	[59.56–70.74]		
Positive	69 (6.61)	30 (10.24)	0.37 (0.27–0.52)	*0*.001*
	[5.26–8.28]	[7.28–14.63]		
Unknown	118 (11.30)	71 (24.23)	0.72 (0.43–1.22)	0.221
	[9.52–13.37]	[19.37–29.46]		
**On Anti-Retroviral Therapy (ART) (n = 87)**				
Yes	38 (84.44)	10 (58.82)	*Ref*	
	[70.54–93.51]	[57.96–86.14]		
No	7 (15.56)	7 (41.18)	3.80 (1.08–13.71)	*0*.038*
	[6.49–29.46]	[13.86–42.04]		
**On Co-trimoxazole preventive Therapy (CPT) (n = 35)**				
Yes	11(91.67)	5 (50.0)	*Ref*	
	[61.52–99.79]	[47.08–86.79]		
No	1 (8.33)	5 (50.0)	11.0 (1.01–120.43)	*0*.05*
	[0.21–38.48]	[13.21–52.92]		

*Statistically significant (p<0.05) R = Reference.

Female patients (OR: 1.41, 95% CI: 1.08–1.85, *p = 0*.*012*), those were attending Private not for Profit (PNFP) facilities (OR: 2.10, 95% CI: 1.57–2.83, *p = 0*.*001*), those diagnosed with Pulmonary TB (OR: 3.66, 95% CI: 1.17–11.43, *p = 0*.*026*), those on Anti-Retroviral Therapy (ART)(OR: 3.66, 95% CI: 1.08–13.71, *p = 0*.*038*), and those on Co-trimoxazole preventive Therapy (CPT) (OR: 11.0, 95% CI: 1.01–120.43, *p = 0*.*05*) had significantly increased odds of 1.41 to 11.0 for successful treatment outcomes compared to the male patients, those were attending Private for Profit (PFP) facilities, those diagnosed with Extrapulmonary TB, those not on ART and those on CPT. Conversely, all age groups (OR: 0.26–0.65, *p = 0*.*001*), those whose TB case were diagnosed 2013 (OR: 0.39, 95% CI: 0.23–0.67, *p = 0*.*001*), those using Genexpert as their means of diagnosis (OR: 0.62, 95% CI: 0.40–0.96, *p = 0*.*031*), and those that are HIV positive (OR: 0.37, 95% CI: 0.27–0.52, *p = 0*.*001*), were shown to have a significantly reduced likelihood for successful treatment outcomes ranging from 38%-74%, compared (Table 2).

### Predictors of TB treatment outcome

When adjusted for possible confounders, multivariate logistics regression model showed a significant higher odds from lower values for Age (AOR: 2.07–2.92, *p = 0*.*001*), year of TB diagnosis (AOR: 2.09, 95% CI: 1.12–3.92, *p = 0*.*012*), means of diagnosis (AOR: 1.72, 95% CI: 1.06–2.77, *p = 0*.*027*), and HIV status (AOR: 2.38, 95% CI: 1.62–3.51, *p = 0*.*001*). Sex and Facility type remained same values (Table 3).

**Table 3:** Factors associated with the number of TB treatments received among the respondents stratified by treatment outcome using multivariate logistic regression.

Variables	COR (95 CI)	*p-value*	AOR (95 CI)	*p-value*
**Age (years) (n = 1656)**				
≤20 ^R^	*Ref*		*Ref*	
21–30	0.26 (0.14–0.47)	*0*.001*	2.92 (1.51–5.65)	*0*.001*
31–40	0.41 (0.24–0.68)	*0*.001*	2.07 (1.16–3.69)	*0*.014*
41–50	0.38 (0.22–0.64)	*0*.001*	2.22 (1.24–4.02)	*0*.01*
51–60	0.37 (0.21–0.66)	*0*.001*	2.47 (1.28–4.76)	*0*.01*
60+	0.65 (0.35–0.66)	*0*.001*	1.37 (0.69–2.75)	*0*.*369*
**Year (n = 1655)**				
2010 ^R^	*Ref*		*Ref*	
2011	0.82 (0.39–1.70)	0.588	0.53 (0.19–1.530	0.241
2012	0.80 (0.46–1.39)	0.419	0.91 (0.48–1.71)	0.759
2013	0.39 (0.23–0.67)	*0*.001*	2.09 (1.12–3.92)	*0*.020*
2014	0.80 (0.51–1.26)	0.338	1.16 (0.68–1.97)	0.585
2015	0.80 (0.51–1.27)	0.340	1.19 (0.69–2.03)	0.522
2016	0.76 (0.48–1.19)	0.228	1.46 (0.87–2.46)	0.152
**Sex (n = 1660)**				
Female			*Ref*	
Male	1.41 (1.08–1.85)	*0*.012*	1.37 (1.02–1.86)	*0*.039*
**Facility Type (n = 1660)**				
Private not for profit (PNFP)			*Ref*	
private for profit (PFP)	2.10 (1.57–2.83)	*0*.001*	2.10 (1.39–2.88)	*0*.001*
**Site of TB (n = 1622)**				
Pulmonary TB			*Ref*	
Extra pulmonary TB	3.66 (1.17–11.43)	*0*.026*	2.24 (0.52–9.68)	*0*.*280*
**Means of diagnosis (n = 1560)**				
Acid-fast bacillus (AFB) ^R^	*Ref*		*Ref*	
Genexpert	0.62 (0.40–0.96)	*0*.031*	1.72 (1.06–2.77)	*0*.027*
Xray	1.31 (0.48–3.56)	0.603	0.78 (0.25–2.41)	0.669
Afb/genexpert	0.70 (0.40–1.22)	0.207	2.11 (1.13–3.96)	*0*.020*
Others	0.86 (0.81–8.05)	0.856	0.78 (0.73–8.37)	0.838
**HIV Status (n = 1614)**				
Negative ^R^	*Ref*		*Ref*	
Positive	0.37 (0.27–0.52)	*0*.001*	2.38 (1.62–3.51)	*0*.001*
Unknown	0.72 (0.43–1.22)	0.221	1.14 (0.62–2.09)	0.669
**On Anti-Retroviral Therapy (ART) (n = 87)**				
Yes			*Ref*	
No	3.80 (1.08–13.71)	*0*.038*	0.21 (0.02–2.89)	0.208
**On Co-trimoxazole preventive Therapy (CPT) (n = 35)**				
Yes			*Ref*	
No	11.0 (1.-120.43)	*0*.05*	0.007 (0.00–0.009)	0.993

*Statistically significant(p<0.05) R = Reference, AOR = Adjusted Odd Ratio, COR = Crude Odd Ratio.

The variables, Site of TB (AOR: 2.24, 95% CI: 0.52–9.68, *p = 0*.*280*), if on ART (AOR: 0.21, 95% CI: 0.02–2.89, *p = 0*.*208*), and if on CPT (AOR: 0.007, 95% CI: 0.00–0.009, *p = 0*.*993*) were confounding determinant of Successful treatment Outcome as they became non-significant at the multivariate analysis level.

**Fig 2:**
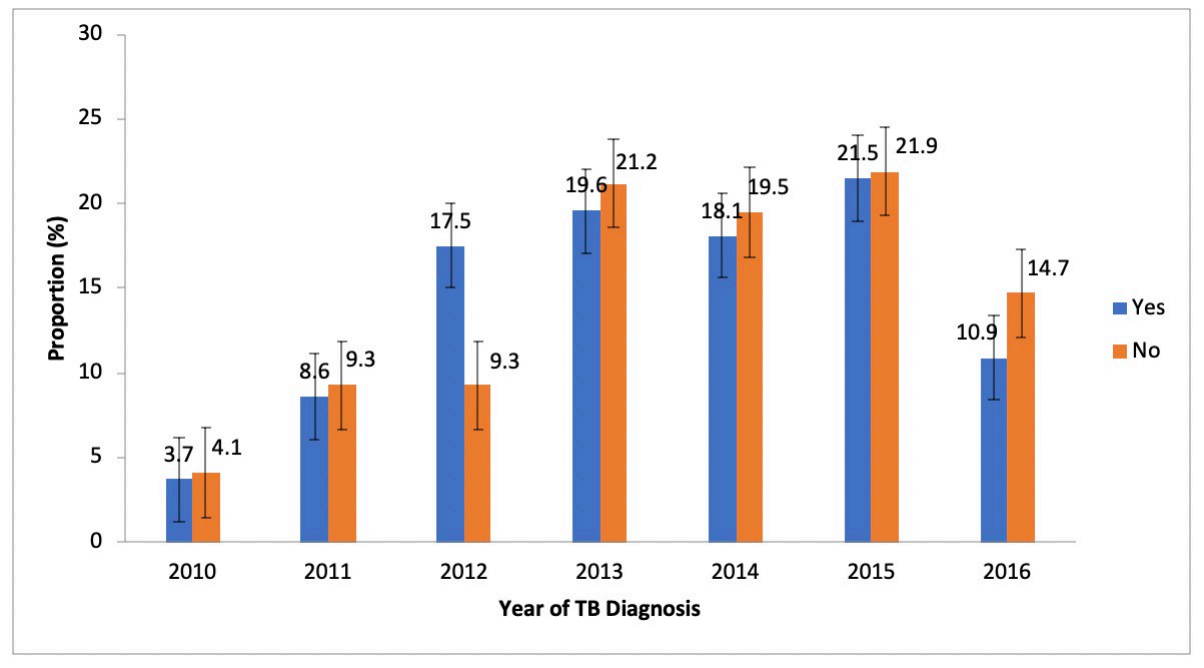
Trend of successful TB treatment outcome (Yes/No) from 2010–2016.

A statistically significant trend of successful TB treatment outcome is observed from 2010 to 2016, with an increased prevalence of 3.7% to 21.5% from 2010 to 2015, and a sharp drop to 10.9% in 2016 *(Chi-Square* ***(χ)***^***2***^ = *110*.*92; p = 0*.001)* as shown in see Fig 2.

## Discussion

The study evaluated trends and predictors of treatment outcome over seven years (2010–2016) among engaged private DOTS providers in Lagos, Nigeria. TSR decreased from 17.7% in 2010–2012 to 10.9% in 2015–2016. The huge proportion of patients that dropped out between 2010–2016 is worrisome and may also imply a reduction in patients’ adherence to medication within that period. Over time, the private health sectors will continue to encounter increased patient load and usually with no designated staff solely for TB DOTS as in the public sector. The private health facilities need to be supported by LSTBLCP on implementation of case holding approaches to improve the disease control. The average seven-year treatment success rate in this study was 78%. This figure is lower than TSR figures of 83.7%, 86%, 85.7% and 83.5% specifically reported from private hospitals in Northern, Southeast and Southwest Nigeria [6,19–21], and but higher when compared to TSR of 75.7% reported from southeastern Nigeria [22] and National pooled estimate of 68.1% reported for six European countries [23]. The study from southeastern Nigeria was conducted in a tertiary setting, a referral centre, and many TB cases referred late, explaining the lower successful treatment outcome when compared with our study [22]. However, TSR in our study was lower than the reported range of 81%-99% from Nigeria, Saudi Arabia, Pakistan, and Ethiopia [24–27]. The lower TSR figures may be due to the high turnover of staff trained on DOTS which was not uncommon with the private sector [28]. Private sector staffs in Nigeria were poorly paid, and they combined multiple clinical and administrative roles in the hospitals; hence the enormous workload facilitated their constant job hunting. In many cases, staff managing TB patients were not trained. Adherence counselling and quality of care often resulted in poor treatment outcomes when TB patients were managed by untrained staff [29–31].

Patients who were managed for TB in 2013 have a lower likelihood of a successful treatment outcome. Variations in odds of treatment outcome across treatment years were similar to findings from Ethiopia [32,33]. TB patients managed in 2013 in our study have lower odds of successful treatment outcomes compared with those treated in 2010. This may be as a result of a significant six-fold increase in the number of TB patients managed in these private facilities between 2010 and 2013 within a limited workforce available in the private sector. This might have hurt the quality of counselling, follow up, and other case holding activities. Increased funding from donors and support from non-governmental organizations to the private clinics resulting in improved training of staff and quality of care could have contributed to the stability in treatment outcomes over subsequent years [8].

Patients diagnosed with genexpert in this study have higher odds of unfavourable TB treatment outcomes compared with those diagnosed with smear. Until 2016, when genexpert became the preferred method of diagnosis for all forms of TB in Nigeria, its use has been limited to suspected TB/HIV, MDRTB, and relapse cases which usually have poorer treatment outcomes [34]. In addition, genexpert was less accessible when compared with smear, and only one facility in our study had a resident Genexpert marchine in the review period. The remaining facilities in this study referred patients’ sputum samples to the distant public facilities for genexpert diagnosis; hence many patients diagnosed with genexpert might have done so late in the course of the disease. In contrast to our finding, a recent metaanalysis highlighted contrasting results with improved treatment outcome two years following the introduction of genexpert [35]. In settings where genexpert is more accessible, there may be a beneficial effect of early identification of TB patients, particularly those with MDRTB, with genexpert (compared with smear) on successful treatment outcomes. The association of genexpert (as compared with smear) on unsuccessful treatment outcomes may, therefore, change when MDR TB patients are the population of interest. One randomized controlled trial (RCT) from Ethiopia among PLHIV on ART demonstrated no effect of Xpert MTB on 3-month mortality when compared with sputum microscopy [36]. Improved outcomes for patients with MDR-TB may be one of the main benefits of Xpert. To maximize the impact of genexpert technology on TB treatment outcome, a rapid point-of-care (POC) TB diagnostics is needed. With TB POC, patients can be diagnosed faster and started on treatment before complications arise.

Age >20 years decreased the likelihood of a successful treatment outcome in this study. This is similar to findings from Ethiopia, Turkey, and Spain [37–40]. Increasing age increases the risk of co-morbidities like diabetes, risk of Lost to Follow Up (LTFU), and accompanied by deterioration in the body’s physiological mechanisms. Previously, increasing age was also associated with reduced access to health services, particularly for the elderly population [39]. This finding could mean that families gave special attention and support needed by younger children to adhere to TB medications. At the same time, older populations took on more family responsibilities and might have found it challenging to adhere to TB medications and keep follow-up appointments [41,42]. Besides, over 30% of patients in our study did not have treatment adherence supporters, leaving patients solely to take responsibility for their health. There is a need for improved adherence support, particularly among older adult TB patients attending private hospitals.

We found significant differences in treatment outcomes between patients who were HIV-negative and HIV-positive. HIV negative patients in this study have lower odds of successful TB treatment outcomes. The findings were similar to those from Nigeria and elsewhere [43–47]. Although 88% of patients in our study were tested for HIV, about 20% of TB/HIV co-infected patients were not on ART which might explain the non-significant association of successful outcome with ART status among TB/HIV co-infected patients. The mechanism leading to unsuccessful treatment outcome among HIV positive TB patients were explained in previous studies [44–46]. The immunosuppression experienced by TB patients as a result of low CD4 count and advanced HIV disease at the time of HIV diagnosis increases the risk of mortality and poor treatment outcome [44–46]. Among TB/HIV co-infected patients in South Africa and Ethiopia, prior studies highlighted the influence of co-morbidities, opportunistic infections and severe malnutrition on mortality as well as severe anemia and comorbidities on treatment failure. This calls for the management of opportunistic infections and co-morbidities among TB/HIV co-infected patients to maximize the benefits of ART initiation [48,49]. The fidelity of TB/HIV collaborative activities, which include 100% HIV testing for TB patients, early initiation of ART for TB patients, as outlined in the NTBLCP guideline, need to be monitored in the private sector. Healthcare workers (HCWs) should be trained to provide quality adherence counselling to reduce the pill burden associated with TB and HIV medications. In one study among TB/HIV co-infected in Ethiopia, patients cited pill burden and perception that their bodies could not tolerate both TB and HIV medications as reasons for defaulting treatment [50].

Point-of-care diagnosis could aid early diagnosis of TB before the onset of complications thereby improving treatment outcomes. By providing clinician with immediate feedback to make informed decisions, non-invasive techniques such as ultrasound are critical tools to reduce morbidity and mortality through early detection of TB in resource limited settings. One such technique described recently is the focused assessment with sonography for HIV-associated TB, FASH protocol [51]. The technique evaluated for pericardial fluid, pleural fluid, ascites, lymphadenopathy, splenic and hepatic lesions and has been shown to detect TB that would be missed by other available diagnostics.Socioeconomic factors influencing outcomes of TB patients managed in private hospitals should be explored in future qualitative studies.

### Strength and limitations

The study was well powered as it reviewed a large sample of routine TB data in private health facilities to evaluate trends and predictors of TB treatment outcome over seven years. It reflected the operational reality for regular clinical data.

Like any retrospective study, some treatment outcome data were incomplete and inadequate, although these were excluded. Variables used for the analysis came from routine TB surveillance data and omitted useful information not captured in the TB register. Such variables might impact directly or indirectly on treatment outcomes like treatment adherence, presence of opportunistic infections (OIs), co-morbidities such as diabetes mellitus, lack of transport fare to the clinic, malnutrition, and adverse events were not included in the study. Also, data on CD4 count for HIV positive patients were not evaluated. We, therefore, recommend prospective studies with these additional details. Its retrospective nature also implied that only clinic records were reviewed while non-clinic data like TB death in the community were not captured.

## Conclusion and recommendation

Treatment outcome among Lagos private hospitals was low compared with NTBLCP and World Health Organization (WHO) target. The huge proportion of patients that dropped out is worrisome and may also imply a reduction in patients’ adherence to medication. Over time, the private sector will continue to encounter increased patient load and usually with no designated staff solely for TB DOTS as in the public health facilities. Promotion of early care-seeking, improving diagnostic and case holding efficiencies of private health facilities, and TB/HIV collaborative interventions to mitigate the risk of an unsuccessful outcome.

We urge the government and TB stakeholders to strengthen the PPM interventions to improve adherence, particularly among People Living with HIV (PLHIV) and older TB patients to improve the health outcome of patients receiving TB treatment at the private health facilities. The private health facilities need to be supported by LSTBLCP on implementation of case holding strategies.

## Supporting information

S1 File (SAV)

## Abbreviations

CD4: Cluster of Differentiation
CPT: Cotrimoxazole Preventive Therapy
DOTS: Directly Observed Therapy Short Course
HIV: Human Immunodeficiency Virus
LSTBLCP: Lagos State Tuberculosis, Buruli Ulcer and Leprosy Control Program
MDRTB: Multidrug-resistant tuberculosis
MOH: Ministry of Health
NTBLCP: National Tuberculosis, Buruli Ulcer and Leprosy Control Program
OIs: Opportunistic Infections
PFP: PrivateFor-Profitt
PHP: Private Health Providers
PLHIV: People Living with HIV
PNFP: Private Not For Profit
POC: Point of Care
PPM: Public-Private Mix
TB: Tuberculosis
TSR: Treatment Success Rate
WHO: World Health Organization
